# Commentary: Misguided Effort with Elusive Implications, and Sifting Signal from Noise with Replication Science

**DOI:** 10.3389/fpsyg.2016.00621

**Published:** 2016-04-29

**Authors:** Martin S. Hagger, Nikos L. D. Chatzisarantis

**Affiliations:** ^1^Health Psychology and Behavioral Medicine Research Group, Faculty of Health Sciences, School of Psychology and Speech Pathology, Curtin UniversityPerth, WA, Australia; ^2^Faculty of Sport and Health Sciences, University of JyväskyläJyväskylä, Finland; ^3^School of Applied Psychology and Menzies Health Institute Queensland, Behavioural Bases for Health, Griffith UniversityBrisbane, QLD, Australia; ^4^School of Human, Health and Social Sciences, Central Queensland UniversityRockhampton, QLD, Australia

**Keywords:** ego-depletion, Self-control, registered replication report, Meta-analysis, experimental fidelity

Driven by its elegant simplicity and intuitive appeal, the ego-depletion effect has received considerable attention in the scientific literature and media. Self-control is conceptualized as a limited resource that becomes depleted after a period of exertion leading to reduced self-control capacity (Baumeister et al., 1998). Our meta-analysis of published studies adopting the sequential-task paradigm to test the ego-depletion effect revealed a medium-sized effect (*d* = 0.62). However, another meta-analysis identified substantive small-study bias and a corrected effect that was close to zero (Carter et al., 2015).

We set out to provide a pre-registered replication of the ego-depletion effect as a *Perspectives on Psychological Science* registered replication report (RRR) to resolve the matter. The depletion RRR adopted a standardized “sequential task” design with a tightly-controlled protocol based on Sripada et al.'s (2014) experiment with 23 laboratories conducting replications. We highlight key findings of the depletion RRR, respond to Baumeister and Vohs' (2016) and Sripada et al.'s (2016) commentaries, highlight some emerging issues, and outline potential ways forward for the field.

Meta-analysis of the RRR findings across labs found the ego-depletion effect to be close to zero corroborating Carter et al.'s analysis (Hagger et al., 2016). Important additional findings from the depletion RRR should be noted: all but one lab predicted a priori that there would be a substantive (non-trivial) effect (so the RRR was not conducted by a group of skeptics), first-spoken language of participants (English-speaking vs. non-English-speaking) did not moderate the effect, there was moderate heterogeneity in the effect across labs, and only three labs found a non-zero effect, including one against the predicted direction.

Baumeister and Vohs raise the issue of “manipulation failure” based on participants' subjective fatigue ratings. While subjective fatigue alone may not adequately capture depletion, the characteristics of tasks used to evoke depletion likely play an important role in determining the effect. Baumeister and Vohs suggest that the depleting task used in the RRR, the letter “e” task, may not have been sufficient to deplete participants because it excluded a “habit forming” period in the depletion condition before the main rule-based regulation period. They claim that without this period there is “nothing to override” and that the task is “contrary to the nature of self-control tasks.” While we have some sympathy with this claim, we do not think it provides sufficient basis to dismiss the task as failing to tax self-control. As we pointed out, participants must suppress the time-pressured urge to respond to any “e” in presented words in favor of the rules—they must stop themselves from making an impulsive judgment as time dictates when they sight an “e” in order to apply the rules. On this basis, we reckon the letter “e” task requires self-control and is consistent with the use of “e-crossing” tasks without the habit-forming period used previously (e.g., Wan and Sternthal, 2008).

Task duration has also been proposed as a reason the RRR failed to evoke depletion. We stress that the tasks adopted in the current paradigm were of similar duration to many previous experiments which have shown depletion effects, including Sripada et al.'s study. We do, however, acknowledge that task duration is an important issue in depletion research. For example, we found duration moderated the depletion effect in our meta-analysis (Hagger et al., 2010). We are particularly interested in task duration, as well as effort on the first task, as a candidate moderator depletion (Lee et al., 2016). The onus is on researchers to develop a clear set of paradigms that reliably evoke depletion in large samples with high power and to systematically explore the effects of moderators like duration and task effort. We also welcome calls by Baumeister and Vohs (2016) and Inzlicht (2016) for further replications using multiple tasks and paradigms, and it is also something we have called for.

Sripada et al. (2016) conducted a Bayesian analysis of replication success on the data from the English-speaking labs of the RRR based on the premise that the effect size was larger than the effect with all labs included (*d* = 0.04 vs. *d* = 0.14). Using their original data as priors, they found that given the data from the RRR, the null hypothesis was more likely in the overall and English-speaking labs, although the probability was lower in the English-speaking labs. However, their claim of a “trend-level” statistically significant effect for the English-speaking labs is somewhat misleading. It does not detract from the fact that the effect for English-speaking labs is small and trivial. To illustrate, the sample size necessary to detect an effect at the *p* = 0.10 level with this effect size, assuming an a priori power of 0.95 would be 1750.

One issue arising from the RRR is the moderate heterogeneity in the effect size across labs, the stringent, clearly specified protocol notwithstanding. Similar levels heterogeneity has been observed in other RRRs (e.g., Eerland et al., 2016). However, Higgins and Thompson (2002) suggest that *I*^2^ values of <40% may be unimportant, and the non-significant Cochran's *Q* values seem to corroborate this view. Nevertheless, one possibility is that even with very strict controls on methods, psychologists in different labs might fail to implement protocols consistently. It may also point to the potential for cultural and interpretive differences in different labs to affect study implementation. Journal editors should encourage researchers to publish clear and precise protocols to facilitate accurate replicability of experimental results. Furthermore, future replications should seek to identify and measure potential moderating variables that may affect study implementation across labs in different countries and contexts.

In conclusion, we feel that the RRR was a fair test of the ego-depletion effect using an accepted paradigm and appropriate tasks. We stress that the RRR is one datum contributing to the debate. For the record, we think that ego-depletion is a “real” phenomenon analogous to cognitive fatigue. But current results suggest that short-term depletion of self-control tested by the sequential task paradigm is problematic. We reiterate our call for high-powered replications of the depletion effect using multiple tasks and a systematic evaluation of candidate moderators.

## Author contributions

MH, NC conceived the ideas in the commentary and drafted the article together.

## Funding

MH contribution on this project was supported by funds from the Finnish Distinguished Professor Programme (FiDiPro) from the Finnish Academy of Science and TEKES and by Discovery Project (#DP130103277) from the Australian Research Council.

### Conflict of interest statement

The authors declare that the research was conducted in the absence of any commercial or financial relationships that could be construed as a potential conflict of interest.
